# Revisiting Postoperative Vision Loss following Non-Ocular Surgery: A Short Review of Etiology and Legal Considerations

**DOI:** 10.3389/fsurg.2017.00034

**Published:** 2017-06-26

**Authors:** Ehud Mendel, Nicoleta Stoicea, Rahul Rao, Weston Niermeyer, Stephen Revilla, Marcus Cluse, Gurneet Sandhu, Gerald J. Todaro, Sergio D. Bergese

**Affiliations:** ^1^Department of Neurological Surgery, The Ohio State University Wexner Medical Center, Columbus, OH, United States; ^2^Department of Anesthesiology, The Ohio State University Wexner Medical Center, Columbus, OH, United States; ^3^West Virginia School of Osteopathic Medicine, Lewisburg, WV, United States; ^4^College of Medicine, The Ohio State University, Columbus, OH, United States; ^5^Arnold Todaro & Welch, Columbus, OH, United States

**Keywords:** ischemic optic neuropathy, malpractice, non-ocular surgery with vision loss, postoperative vision loss, neurosurgery

## Abstract

Postoperative vision loss (POVL) following non-ocular surgery is a serious complication where the causes are not fully understood. Studies have identified several causes of POVL as well as risk factors and prevention strategies. POVL research is made difficult by the fact that cases are often subject to malpractice claims, resulting in a lack of public access to case reports. This literature review was conducted in order to identify legal issues as a major barrier to studying POVL and address how this affects current knowledge. Informed consent provides an opportunity to overcome legal challenges by reducing malpractice litigation through educating the patient on this outcome. Providing pertinent information regarding POVL during the informed consent process has potential to reduce malpractice claims and increase available clinical information.

## Introduction

**Figure 1:**
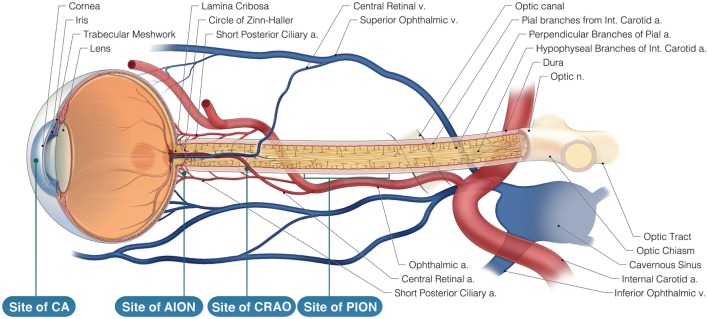
Neurovasculature of the eye. Arterial supply to the optic nerve and the retina comes from branches of the internal carotid artery. The retina is solely supplied by the central retinal artery. Veins of the retina drain into the cavernous sinus. AION is located anteriorly to the lamina cribosa and is most likely caused by posterior ciliary artery occlusion while Posterior ION (PION) is posterior and results from improper pial vessel supply. Central retinal artery occlusion (CRAO) is a result of emboli and globe compression resulting in a loss of blood supply of the surface layer of the optic disk. Corneal abrasion (CA) is due to inhibition of corneal reflex and decreased tear production.

Postoperative vision loss (POVL) during non-ocular procedures is a devastating complication following surgery under general anesthesia. There is significant variation in the reported incidence of POVL ranging from 0.056 to 1.3% (1). Surgical procedures posing the highest risk for POVL are cardiac (incidence = 0.09%) and spinal surgeries (incidence as high as 0.2%) (2, 3). Etiologies of POVL include ischemic optic neuropathy (ION), central retinal artery occlusion (CRAO), cortical blindness (CB), and corneal abrasion (CA) (4). Strong evidence indicates an increasing incidence of POVL in part due to the rising number of spinal fusion operations (500% from 1993 through 2004) (5). Risk factors for these conditions have been identified; however, the mechanisms are not yet fully understood (4, 6–8). Male gender, prone position during surgery, hypotension, prolonged procedures, longer anesthesia duration, and decreased use of colloids are associated with POVL (9–13). Certain modifiable risk factors are obesity, cardiovascular disease, hyperlipidemia, diabetes mellitus, and smoking (4, 6, 7, 14). We intend to review the medicolegal barriers preventing access to current POVL information and subsequently hindering the advancement of knowledge on this topic (Figure 1).

## Literature Search Methodology

A literature search was conducted *via* PubMed, Google Scholar, and American Society of Anesthesiologists (ASA) Closed Claims Databases using the following keywords: *postoperative vision loss, ischemic optic neuropathy, malpractice*, and *non-ocular surgery* with *vision loss*. Articles within the search criteria included case reports, registry reports, reviews, randomized trials, cohort studies, newsletters, and case control studies published since 2000. Non-English literature was excluded from the search.

## Results/Discussion

Vision loss caused by non-ocular surgery poses severe implications for a patient’s quality of life. POVL is frequently involved in malpractice claims complicating the study of its mechanisms and assessment of causative factors (15). Until legal action is resolved, reporting POVL cases can be delayed up to 7 years (16). Although there is no mandated reporting system for POVL, the ASA Closed Claims Project registry was created in 1999 to facilitate physicians’ access to an up-to-date database of vision loss cases after non-ophthalmic surgery (17). In 2014, the project database contained 10,093 claims of which 7,351 were surgical anesthesia claims (18). Non-ocular POVL cases are submitted to the registry without patient, provider, or institutional identifiers in order to protect confidentiality and encourage hospitals to report events of POVL with minimal legal ramifications (19). ASA members are able to request access to search the database; however, such requests are limited to “simple analysis of narrowly defined topics.” (17) Cases submitted to the registry remain protected from public access (17). In 2014, the Agency for Healthcare Research and Quality reported that plaintiff attorneys made numerous requests for release of the POVL registry data to the public. The registry denied public release of the information in accordance with board-approved confidentiality procedures (19). The rarity of this complication and the complexity of reported cases have resulted in a paucity of research on POVL. Because current data are limited, there is no well-established standard of care. The ASA Perioperative Visual Loss Task Force developed a practice advisory focused on perioperative management for patients identified to be at high risk (20). Without a complete understanding of this serious adverse event, advances in establishing POVL management guidelines are hindered.

Research into the underlying pathophysiology of more common causes of POVL, such as CA, has led to more effective prevention strategies and fewer malpractice claims (1, 21, 22) (Table 1).

**Table 1:** Summary of identified causes of postoperative vision loss (POVL) and malpractice claims.

Identified cause of POVL	Pathophysiology	Clinical presentation	Incidence range	Postoperative eye injury malpractice claim incidence (1980–2011)	Permanent eye injuries (1980–2011)	Median claim payment (1980–2011)
Corneal abrasion	Decreased corneal protection through inhibition of corneal reflex and decreased tear production (21)	Complaints of blurry vision, tearing, redness, photophobia, foreign body sensation (23)	0.17–44% during the perioperative period (23)	31% (1980–1994) 18% (1995–2011) (18)	49% (1980–1994) 73% (1995–2011) (24)	$128,100 (1980–1994) $424,750 (1995–2011) (24)
Ischemic optic neuropathy	Not well understood; proposed mechanisms include increased intraocular pressure and ophthalmic vein congestion (25)	AION: painless and progressive deterioration of vision, optic disk edema which resolves spontaneously in 7.9–11.4 weeks	89% of POVL occurring from spine surgery; Posterior ION (PION) accounts for 60% of these cases (26)	**Optic nerve injuries:** 5% (1980–1994) 38% (1995–2011) (24)		
		PION: acute painless visual loss in one or both eyes that can progress to complete blindness (14)				
Central retinal artery occlusion	Emboli and direct pressure on the globe (9)	Typically manifests unilaterally with “cattle tracking” of the arterioles with a “cherry-red” spot visible during fundoscopic exam (27)	11% of spine surgeries (26)			
Cortical blindness (CB)	Ischemia or extreme hypoperfusion of the occipital lobes (7)	Deteriorating vision that results in partial or bilateral POVL (7)	0.0038% of POVL cases due to CB (28)	–		

### Corneal Abrasion

Corneal abrasion while under general anesthesia is a result of decreased corneal protection through inhibition of the corneal reflex and decreased tear production (22). Longer procedures, as well as prone or lateral positioning during surgery, are shown to increase the risk of CA (21). Many institutions have developed prevention strategies for CA (18). Treatment of CA usually involves administration of artificial tears and antibiotic ointment (23). No intervention is completely effective in protection of CA during surgery, but eyelid taping is the single best used protective method. Proper placement of eyelid protection has been shown to decrease the risk of CA (21). CA claims from 1990 or later received a median compensation of $12,000. The registry reports a decrease in the incidence of claims from 31% in 1980–1994 to 18% in 1995–2011, most of them being associated with general anesthesia (18). The reduction in CA malpractice claims from 1980 to 2011 is likely reflective of the effective prevention measures taken by anesthesiologists prior to surgery.

Permanent eye injury following non-ocular surgery, a less understood complication with severe patient outcomes, is a vital area for further research often delayed by malpractice claims (24). Lee et al. reported that permanent eye injury claims and optic nerve damage claims increased from 1980 to 2011 with the majority of closed claims involving spinal surgeries (24). Permanent injury following surgery increased from 49 to 73% of total reported eye injury cases. The median payment made for permanent vision loss malpractice claims escalated from $128,100 (1980–1994) to $424,750 (1995–2011), when adjusted for 2013 inflation (24).

### Ischemic Optic Neuropathy

Ischemic optic neuropathy is the most common cause of permanent POVL after non-ocular surgery (26, 29). Prone and Trendelenburg positioning during surgery can increase intraocular pressure (IOP) and cause ophthalmic vein congestion leading to ION, and in some cases, permanent POVL (25, 29). The ASA Postoperative Visual Loss Registry reported that 89% of perioperative vision loss in spine surgery is due to ION, with posterior ION (PION) accounting for 60% of cases (26).

A recent article published by Rubin et al. studied trends in ION incidence in spinal fusion using a large nationwide hospital database from 1998 to 2012 and concluded that perioperative ION in spinal fusion decreased by 2.7-fold. Aging, obesity, male gender, and transfusions were significantly associated with ION (30).

Posterior ION is most commonly associated with operations performed in the prone position and of longer duration, and typically presents as painless loss of vision when the patient awakens from anesthesia (29). While anemia and hypotension are observed in these patients, the exact mechanism by which the ischemia occurs remains unclear. Although predisposing factors have been identified, no single causative mechanism for ION can fully explain the etiology under various surgical circumstances (3). Quraishi et al. published a case where improved vision after surgery in a patient diagnosed with PION was achieved by managing hemoglobin, hematocrit, and systolic blood pressure in order to adequately maintain ocular perfusion (31). Hassani et al. demonstrated that administering recombinant human erythropoietin reversed the effects of PION following a 6 h procedure with significant blood loss (32). The ASA Task Force on Perioperative Visual Loss reports that no complete treatment guideline has been established. The recommendation is that consideration should be given to informing the patient of the “small” and “unpredictable risk” of POVL in high-risk cases and to employ preventative strategies (33).

When the patient notices even slight changes in vision, a thorough physical exam should be performed as the viability of POVL treatment options tend to decline over time. Contractor and Hardman stated that all patients experiencing signs of POVL require urgent review by an ophthalmologist (34).

### Central Retinal Artery Occlusion

Central retinal artery occlusion accounts for a small percentage of POVL cases, but is the second most common cause associated with spinal surgery (5). According to the ASA POVL registry, POVL in 11% of spine surgery cases is caused by CRAO (3, 26). CRAO is commonly associated with emboli and direct compression of the globe (9). Unlike PION, CRAO more commonly manifests unilaterally. Prone-positioned surgery increases the risk of CRAO due to external ocular compression produced by the weight of the head against the headrest (5, 15, 33). CRAO is considered reversible if treated within 6 h (35). Treatments including vasodilators, ocular massage, and thrombolytic agents are able to improve visual deficits caused by CRAO, but their effects are poorly demonstrated in ION (22).

### Cortical Blindness

Cortical blindness is known as the third major identified cause of POVL in non-ocular surgery. CB is a loss of vision caused by ischemia or extreme hypoperfusion of the occipital lobes. This can manifest as bilateral vision loss ranging from partial to complete (7). Most cases of CB are caused by spontaneous ischemic stroke (32%), cardiac surgery (20%), and cerebral angiography (12%) (36).

Intraoperative factors, such as anesthetic duration, blood loss, position during surgery, and fluid administration, are equally important to consider. The ASA Practice Advisory states that patients should be positioned with their head at the same level or higher than the heart and maintained in a neutral forward position when possible in order to reduce IOP and prevent POVL from occurring (33). The ASA POVL Registry data collected between 1999 and 2012 shows that 94% of ION cases resulted from surgeries performed under general anesthesia for 6 h or longer. It has been suggested that procedures requiring prolonged anesthesia can be staged in order to reduce length of the surgery. If staging is to be used, the patient should be informed of the risk imposed by this type of procedure compared to prolonged anesthesia (33). Although these possible strategies have been suggested, there is no evidence to fully support their use in preventing ION (37).

Due to the different etiologies of POVL and the limited effectiveness of treatments, disclosure of information regarding POVL prior to surgery is appropriate to consider (38). One method to accomplish this disclosure is through informed consent. The case *Salgo v Stanford* (1957) first used the term *informed consent* and ruled that informed consent should make known to patients all potential risks, benefits, and alternative treatments before undergoing surgical or anesthetic procedures (38–40). The court’s decision states, “a physician violates his duty to his patient and subjects himself to liability if he withholds any facts … necessary to form the basis of an intelligent consent by the patient to the proposed treatment” (40). Furthermore, the court concluded that physicians were permitted to use discretion in order to withhold facts based upon the patient’s mental and emotional state (39). In the early 1970s, a new version of informed consent doctrine quickly became recognized “in one form or another in virtually every state.” This doctrine dictated that, in addition to giving a patient the decision to consent to or refuse healthcare, a physician was required to provide the patient with all information that a “reasonably prudent person” would find “material” to making this decision (39). One survey published in 2011 determined that out of 437 patients undergoing spinal surgery at Mayo Clinic in Florida, 80% preferred full disclosure of the risks of POVL (38). During an Anesthesia Patient Safety Foundation conference, a consensus was reached regarding discussion of POVL due to ION during the informed consent process of patients considered to be at risk by anesthesiologists and surgeons (41). The informed consent process may further advise patients on occurrence of POVL, risk factors both modifiable and non-modifiable, and prevention strategies without eliminating the risk of developing POVL (41).

## Conclusion

Patient-centered guidelines on discussing POVL occurrence, risk factors, and prevention strategies are essential. The rare incidence of POVL and the resulting paucity of publications account for the limited understanding of this complication. Bolstering the data available to physicians and researchers could facilitate better knowledge of POVL pathophysiology, risk factors, and contribute to the development of preemptive measures and treatment techniques to improve patient outcomes.

## Author Contributions

RR, WN, SR, and MC conducted the literature search and participated in early drafting of the manuscript. EM, NS, SB, and GS participated in drafting of the article and provided critical review of the clinical information. GT provided insight and guidance for the legal sections of the manuscript. NS conducted the final revision of the manuscript. This project was performed under the guidance and oversight of SB.

## Conflict of Interest Statement

The authors declare that the research was conducted in the absence of any commercial or financial relationships that could be construed as a potential conflict of interest.

## References

[1] Kitaba A Martin DP Gopalakrishnan S Tobias JD. Perioperative visual loss after nonocular surgery. J Anesth (2013) 27(6):919–26. 10.1007/s00540-013-1648-y 23775280

[2] Goni V Tripathy SK Goyal T Tamuc T Panda BB Shashidhar BK. Cortical blindness following spinal surgery: very rare cause of perioperative vision loss. Asian Spine J (2012) 6(4):287–90. 10.4184/asj.2012.6.4.287 23275814 PMC3530705

[3] Nickels TJ Manlapaz MR Farag E. Perioperative visual loss after spine surgery. World J Orthop (2014) 5(2):100–6. 10.5312/wjo.v5.i2.100 24829872 PMC4017302

[4] De la Garza-Ramos R Samdani AF Sponseller PD Ain MC Miller NR Shaffrey CI Visual loss after corrective surgery for pediatric scoliosis: incidence and risk factors from a nationwide database. Spine J (2016) 16(4):516–22. 10.1016/j.spinee.2015.12.031 26769351

[5] Baig BN Lubow M Immesoette P Bergese SD Hamdy E Mendel E Vision loss after spinal surgery: review of the literature and recommendations. Neurosurg Focus (2007) 23(5):E15 10.3171/FOC-07/11/15 18004963

[6] Li A Swinney C Veeravagu A Bhatti I Ratliff J. Postoperative visual loss following lumbar spine surgery: a review of risk factors by diagnosis. World Neurosurg (2015) 84(6):2010–21. 10.1016/j.wneu.2015.08.030 26341434

[7] Yu HD Chou AH Yang MW Chang CJ. An analysis of perioperative eye injuries after nonocular surgery. Acta Anaesthesiol Taiwan (2010) 48(3):122–9. 10.1016/S1875-4597(10)60043-4 20864060

[8] Uribe AA Baig MN Puente EG Viloria A Mendel E Bergese SD. Current intraoperative devices to reduce visual loss after spine surgery. Neurosurg Focus (2012) 33(2):E14. 10.3171/2009.8.FOCUS09151 22853832

[9] Lee J Chin JH Koh WU Ro YJ Yang HS. Unilateral postoperative visual loss in a patient undergoing hip arthroscopy in the supine position: a case report. Korean J Anesthesiol (2016) 69(2):197–9. 10.4097/kjae.2016.69.2.197 27066213 PMC4823420

[10] Mukherjee B Alam MS. Acute visual loss with ophthalmoplegia after spinal surgery: report of a case and review of the literature. Indian J Ophthalmol (2014) 62(9):963–5. 10.4103/0301-4738.143951 25370405 PMC4244749

[11] Patel AV Desai AK Vala RH Patel EM Unilateral vision loss after prolonged prone position in spinal surgery. IJHSR (2015) 5(1):369–73.

[12] Lee LA. Risk factors associated with ischemic optic neuropathy after spinal fusion surgery. Anesthesiology (2012) 116(1):15–24. 10.1097/ALN.0b013e31823d012a 22185873

[13] Roth S Tung A Ksiazek S. Visual loss in a prone-positioned spine surgery patient with the head on a foam headrest and goggles covering the eyes: an old complication with a new mechanism. Anesth Analg (2007) 105(5):1185–7. 10.1213/01.ane.0000264319.57758.55 17456671

[14] Hayreh SS. Management of ischemic optic neuropathies. Indian J Ophthalmol (2011) 59(2):123. 10.4103/0301-4738.77024 21350282 PMC3116541

[15] Roth S Barach P Postoperative visual loss: still no answers – yet. Anesthesiology (2001) 95(3):575–7. 10.1097/00000542-200109000-00006 11575526

[16] Molloy BL. Implications for postoperative visual loss: steep Trendelenburg position and effects on intraocular pressure. AANA J (2011) 79(2):115–21. 21560974

[17] Postoperative Visual Loss Registry. Anesthesia Closed Claims Project. (2016). Available from: http://depts.washington.edu/asaccp/projects/postoperative-visual-loss-registry

[18] Posner KL Lee LA Anesthesia malpractice claims associated with eye surgery and eye injury: highlights from the anesthesia closed claims project data request service. ASA Newsl (2014) 78(11):28–30.

[19] Gliklich RE Dreyer NA Leavy MB, editors. Registries for Evaluating Patient Outcomes: A User’s Guide. 3rd ed Rockville, MD: Agency for Healthcare Research and Quality (US) (2014). Protecting Data: Confidentiality and Legal Concerns of Providers, Manufacturers, and Health Plans.

[20] Newman NJ. Perioperative visual loss after nonocular surgeries. Am J Ophthalmol (2008) 145(4):604–10. 10.1016/j.ajo.2007.09.01 18358851 PMC2989384

[21] Grixti A Sadri M Watts MT. Corneal protection during general anesthesia for nonocular surgery. Ocul Surf (2013) 11(2):109–18. 10.1016/j.jtos.2012.10.003 23583045

[22] Stevens G Prone to blindness: answers to postoperative visual loss. Anesth Analg (2011) 12(1):11–2. 10.1213/ANE.0b013e3181fe772d 21173205

[23] Segal KL Fleischut PM Kim C Levine B Faggiani SL Banerjee S Evaluation and treatment of perioperative corneal abrasions. J Ophthalmol (2014) 2014:Article ID 901901 10.1155/2014/901901 PMC3941207 24672709

[24] Lee L Posner KL Domino KB Trends in injuries to the visual pathways and medicolegal payments from the closed claims project database. Anesthesiology (2013):A2058.

[25] Farag E Cheung C Kurz A Recent advances in understanding the causes of postoperative vision loss after spine surgery. J Anesth Clin Res (2012) 3:e105 10.4172/2155-6148.1000e105

[26] Lee LA Roth S Posner KL Cheney FW Caplan RA Newman NJ The American Society of Anesthesiologists Postoperative Visual Loss Registry: analysis of 93 spine surgery cases with postoperative visual loss. Anesthesiology (2006) 105(4):652–9. 10.1097/00000542-200610000-00007 17006060

[27] Varma DD Cugati S Lee AW Chen CS. A review of central retinal artery occlusion: clinical presentation and management. Eye (2013) 27:688–97. 10.1038/eye.2013.25 23470793 PMC3682348

[28] Shen Y Drum M Roth S. The prevalence of perioperative visual loss in the United States: a 10-year study from 1996 to 2005 of spinal, orthopedic, cardiac, and general surgery. Anesth Analg (2009) 109(5):1534–45. 10.1213/ane.0b013e3181b0500b 19713263

[29] Kla KM Lee LA. Perioperative visual loss. Best Pract Res Clin Anaesthesiol (2016) 30:69–77. 10.1016/j.bpa.2015/11/004 27036604

[30] Rubin DS Parakati I Lee LA Moss HE Joslin CE Roth S. Perioperative visual loss in spine fusion surgery: ischemic optic neuropathy in the United States from 1998 to 2012 in the nationwide inpatient sample. Anesthesiology (2016) 125(3):457–64. 10.1097/ALN.0000000000001211 27362870 PMC5270754

[31] Quraishi NA Wolinsky JP Gokaslan ZL. Transient bilateral post-operative visual loss in spinal surgery. Eur Spine J (2012) 21(4):495–8. 10.1007/s00586-011-2117-7 22170448 PMC3369061

[32] Hassani V Homaei MM Shahbazi A Zamani MM Safari S Nadi S Human erythropoietin effect in postoperative visual loss following spine surgery: a case report. Anesth Pain Med (2014) 4(2):e7291. 10.5812/aapm.7291 24790903 PMC3997951

[33] American Society of Anesthesiologists Task Force on Perioperative Visual Loss. Practice advisory for perioperative visual loss associated with spine surgery: an updated report by the American Society of Anesthesiologists Task Force on Perioperative Visual Loss. Anesthesiology (2012) 116(2):274 10.1097/ALN.0b013e31823c104d 22227790

[34] Contractor S Hardman JG Injury during anaesthesia. Contin Educ Anaesth Crit Care Pain (2006) 6(2):67–70. 10.1093/bjaceaccp/mkl004

[35] Cugati S Varma DD Chen CS Lee AW. Treatment options for central retinal artery occlusion. Curr Treat Options Neurol (2013) 15(1):63–77. 10.1007/s11940-012-0202-9 23070637 PMC3553407

[36] Aldrich MS Alessi AG Beck RW Gilman S. Cortical blindness: etiology, diagnosis, and prognosis. Ann Neurol (1987) 21(2):149–58. 10.1002/ana.410210207 3827223

[37] Roth S. Perioperative visual loss: what do we know, what can we do? Br J Anaesth (2009) 103(Suppl 1):i31–40. 10.1093/bja/aep295 20007988 PMC2791856

[38] Corda DM Dexter F Pasternak JJ Trentman TL Brull SJ Nottmeier EW. Patients’ perspective on full disclosure and informed consent regarding postoperative visual loss associated with spinal surgery in the prone position. Mayo Clin Proc (2011) 86(9):865–8. 10.4065/mcp.2011.0279 21878598 PMC3258003

[39] Dolgin JL The legal development of the informed consent doctrine: past and present. Camb Q Healthc Ethics (2010) 19(01):97–109. 10.1017/S0963180109990284 20025806

[40] Salgo v. Leland Stanford etc. Bd. Trustees P. 2d. (Vol. 317). (1957). 170 p. Cal: Court of Appeals, 1st Appellate Dist, 1st Div.

[41] Lee LA Stoelting RK APSF-sponsored conference on perioperative visual loss develops consensus conclusions. APSF Newslett (2013) 27(3):52.

